# Assessment of mitigation strategies as tools for risk management under future uncertainties: a multi-model approach

**DOI:** 10.1007/s11625-017-0521-6

**Published:** 2018-01-08

**Authors:** Shunsuke Mori, Toyoaki Washida, Atsushi Kurosawa, Toshihiko Masui

**Affiliations:** 1 Tokyo University of Science, Yamasaki 2641, Noda, Chiba 278-8510 Japan; 2 Sophia University, Kioi-cho, 7-1, Chiyoda-ku, Tokyo, 102-0094 Japan; 3 The Institute of Applied Energy, Shinbashi SY Bldg., Nishishnbashi 1-14-2, Minato-ku, Tokyo, 105-0003 Japan; 4 National Institute for Environmental Studies, Onogawa 16-2, Tsukuba, Ibaraki 305-0053 Japan

**Keywords:** Climate change, Risk management, Integrated assessment model, Multi-model approach, Meta-analysis

## Abstract

Although the world understands the possible threat of the future of climate changes, there remain serious barriers to be resolved in terms of policy decisions. The scientific and the societal uncertainties in the climate change policies must be the large part of this barrier. Following the Paris Agreement, the world comes to the next stage to decide the next actions. Without a view of risk management, any decision will be “based on neglecting alternatives” behavior. The Ministry of the Environment, Japan has established an inter-disciplinary research project, called Integrated Climate Assessment—Risks, Uncertainties, and Society (ICA-RUS) conducted by Dr. Seita Emori, National Institute for Environmental Studies. ICA-RUS consists of five research themes, i.e., (1) synthesis of global climate risks, (2) optimization of land, water, and ecosystem for climate risks, (3) analysis of critical climate risks, (4) evaluation of climate risk management options, and (5) interactions between scientific and social rationalities. We participated in the fourth theme to provide the quantitative assessment of technology options and policy measures by integrating assessment model simulations. We employ the multi-model approach to deal with the complex relationships among various fields such as technology, economics, and land use changes. Four different types of integrated assessment models, i.e., MARIA-14 (Mori), EMEDA (Washida), GRAPE (Kurosawa), and AIM (Masui), participate in the fourth research theme. These models contribute to the ICA-RUS by providing two information categories. First, these models provide common simulation results based on shared socioeconomic pathway scenarios and the shared climate policy cases given by the first theme of ICA-RUS to see the ranges of the evaluation. Second, each model also provides model-specific outcomes to answer special topics, e.g., geoengineering, sectoral trade, adaptation, and decision making under uncertainties. The purpose of this paper is to describe the outline and the main outcomes of the multi-model inter-comparison among the four models with a focus upon the first and to present the main outcomes. Furthermore, in this study, we introduce a statistical meta-analysis of the multi-model simulation results to see whether the differently structured models provide the inter-consistent findings. The major findings of our activities are as follows: First, in the stringent climate target, the regional economic losses among models tend to diverge, whereas global total economic loss does not. Second, both carbon capture and storage (CCS) as well as BECCS are essential for providing the feasibility of stringent climate targets even if the deployment potential varies among models. Third, the models show small changes in the crop production in world total, whereas large differences appear between regions. Fourth, the statistical meta-analysis of the multi-model simulation results suggests that the models would have an implicit but common relationship between gross domestic product losses and mitigation options even if their structures and simulation results are different. Since this study is no more than a preliminary exercise of the statistical meta-analysis, it is expected that more sophisticated methods such as data mining or machine learning could be applicable to the simulation database to extract the implicit information behind the models.

## Introduction

### Background of the ICA-RUS project

Although the world widely understands the possible threat of future climate changes, serious barriers that surround policy decisions need to be resolved. The scientific and societal uncertainties in climate change policies are likely a large part of this barrier. After a long debate, the scientific community has concluded in IPCC-AR5 (WG-I SPM 2013) that “human influence on the climate system is clear. This is evident from the increasing greenhouse gas concentrations in the atmosphere, positive radiative forcing, observed warming, and understanding of the climate system.” After considering the above quote, the Paris Agreement is taken into effect on November 2016 after long-term negotiations. The target of this agreement is to limit the atmospheric temperature rise to 2.0 °C, or more preferably 1.5 °C, over pre-industrial levels. Future climate change, its impact on the human society, and the human behavior are still very uncertain; however, the governments of the world need to make decisions by considering the possible damages caused by climate changes as well as their costs. Without the viewpoint of risk management, any decision will be equivalent to “neglecting alternatives” behavior.

Over the past decades, many technological developments with respect to less or zero carbon emission energy options and policy instruments, such as carbon tax and emission trading, have been proposed. Currently, technological proposals have spread from low-cost renewables to geoengineering and satellite power stations. Note that the world presently has no universal and practical option that is currently available to solve the issue of climate change. In other words, the global community needs to find the “most hopeful” portfolio for the future among its options rather than devoting itself to the development of a single option. To establish such a portfolio, we need to address the policy options and technological strategies, including adaptation options and geoengineering under uncertainty. Then, we need to quantitatively assess the costs, benefits, and risks of possible actions. Previously, the only tool to address this subject was the integrated assessment model (IAM), even though no standardized method has yet been established. However, the studies from the views of risk management, especially for the strategy of the “limiting under 1.5 °C” society, have not been discussed. Reflecting the above background, the world climate community has developed the Shared Socioeconomic Pathways (SSPs) to deal with future societal possibilities including population, gross domestic product (GDP), energy, land use, and greenhouse gas emissions identifying five different future scenarios (Riahi et al. 2017; Kriegler et al. 2017).

Accordingly, The Ministry of the Environment, Japan established an inter-disciplinary research project, Integrated Climate Assessment Risks, Uncertainties, and Society (ICA-RUS), which was conducted by Dr. Seita Emori of the National Institute for Environmental Studies for the period of 2012–2016. The purpose of the ICA-RUS project is to provide a basis for the social deliberation on long-term climate goals by exploring advantages and disadvantages involving different targets from a risk management perspective. It attempts to integrate insights from areas of climate risk assessment, energy economics modeling, the energy–water–food–ecosystem nexus, and science and technology studies. ICA-RUS consists of three steps, namely, Step-1 to define the mitigation target including 1.5 and 2.0 °C and Step-2 to assess the consequences and their ranges, where climatic, mitigative, and socioeconomic uncertainties are dealt with. ICA-RUS and Step-3 consider the possibilities of such alternative options as adaptation and geoengineering, including solar radiation management. As a risk management framework, ICA-RUS involves climate science, engineering, economics, and sociology fields to integrate the climate change impacts, mitigation options, and societal acceptance of stakeholders. Thus, ICA-RUS constitutes an inter-disciplinary research project.

**Fig. 1:**
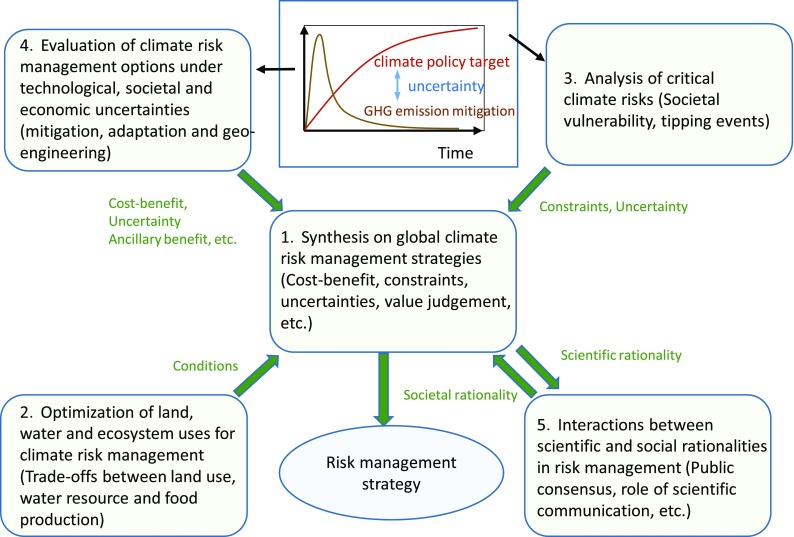
Outline of the ICA-RUS project. (NIES 2014)

Figure 1 shows the outline of ICA-RUS, which comprises five research themes. Theme 1 primarily provides latest information from the field of climate science. Theme 2 and Theme 3 examine the impacts of climate change on the biosphere and on agriculture and critical risks, respectively. Theme 4 focuses on quantitative assessment of technology options and policy measures to limit warming to 2.5, 2.0, and 1.5 °C from the viewpoints of economic impacts, energy supply and demand structures, technologies, and land use changes considering uncertainties. Theme 5 addresses societal attitudes toward risk management from a sociological viewpoint.

The present study is related to Theme 4. We employ the multi-model approach to deal with the complex relationships among fields. Four different types of IAMs participate in this activity. These models provide (1) common simulation results based on the SSP scenarios and the shared climate policy cases given by the first theme of ICA-RUS to determine the evaluation ranges and also (2) model-specific outcomes. In this paper, we focus on the first one and describe the main outcomes of the multi-model inter-comparison among the four models. Furthermore, we introduce a statistical meta-analysis of the multi-model simulation results to see whether the differently structured models provide the inter-consistent findings.

The structure and further details of the entire ICA-RUS project will be described in other papers (NIES 2013, 2014, 2015; Emori et al. 2017).

### Multi-model approach

Integrated assessment model (IAMs) were developed in the early stages of climate change studies mainly to evaluate mitigation costs. Pioneering work on IAMs in this field was conducted by the Edmonds–Reilly model (Edmonds and Reilly 1983) used in the IPCC First Assessment Report. The DICE model (Nordhaus 1992) and the GLOBAL 2100 model (Manne and Richels 1992) were also used in the early development of IAMs as nonlinear optimization formulations. These two models are being expanded in various ways to reflect new scientific findings. For example, DICE has been modified to evaluate global, regional, and ultra-long-term climate policies (Nordhaus 2016) while maintaining its fundamental structure. Conversely, to comprehensively assess global warming mitigation options, several energy–economy models have been expanded to include other fields such as land use changes, food supply–demand models, climate models, and water resources. Therefore, IAMs have often evolved from a single model to multi-module projects. Some examples include AIM (Fujimori 2012), MESSAGE (Messner and Strubegger 1995; IIASA 2017a), GCAM (Edmonds et al. 1997; Clarke et al. 2007; Calvin et al. 2012), MiniCAM (Brenkert et al. 2003), MIT-EPPA (EPPA 2017; Kim et al. 2017), WITCH (Bosetti and Zwaan 2009; FEEM 2011), IMAGE-2 (Strengeers 2001), REMIND-MAgPIE (Kriegler et al., 2017), MERGE (Manne et al. 1993; Clarke et al. 2007), and GRAPE (Kurosawa 2006). IPCC-AR5-WG3 involves 30 IAMs (IPCC 2014a) to generate ranges for climate policies.

**Fig. 2:**
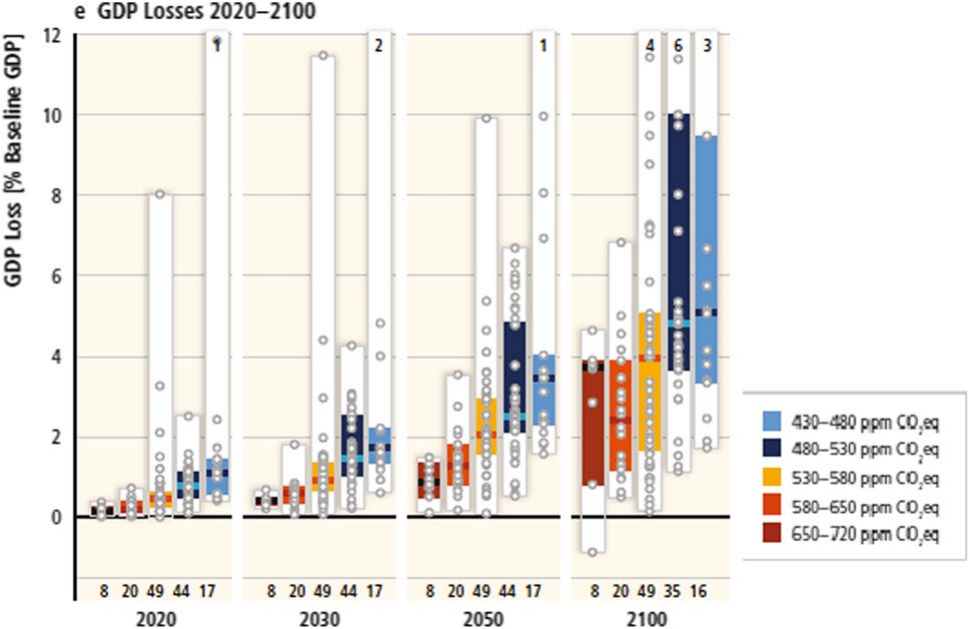
Model simulation distribution for GDP losses under idealized implementation scenarios (IPCC-AR5-WG3 2014b)

Note that the above IAM development activities provide various simulation results under certain climate scenarios that hardly seem to converge, especially under stringent climate control policy, as shown in Fig. 2.

The above observation suggests that simulation results from a single model are insufficient to reach a robust conclusion. When considering the broad ranges and uncertainties in climate science, technological development, economic behavior, and policy and societal phenomena, no one expects a single model to be able to integrate current knowledge and information. Therefore, model researchers understand the need for multi-model inter-comparisons to extract agreed-on and diverging findings under consistent assumptions to provide robust policy recommendations to decision makers. The Energy Modeling Forum (EMF) established by Stanford University in 1976 (EMF 2017) is the leader in multi-model comparisons in this field. The Special Report on Emission Scenarios (IPCC 2000) extensively used this approach, with six IAMs providing simulation results under the same future societal scenarios for population, GDP, final energy demand, and CO_2_ emissions. In the twenty-first century, as various institutes and organizations have developed integrated assessment projects, other model inter-comparison projects have been established (ADVANCE 2017; IDDRI 2017). Nine model inter-comparison projects are involved in Table A.II.15 of IPCC-AR5-WG3 (2014c). The climate model community also established the coupled model inter-comparison project (CMIP) in 1995 (CMIP 2017).

On the basis of the context given above, the ICA-RUS project also utilizes a multi-model approach. Note that although the divergence of the economic losses under the stringent climate policy (as shown in Fig. 2) has been often pointed out, further analysis on the relationship between economic loss and other key drivers has not been carried out in the existing inter-model comparison projects. The meta-analysis approach among model simulations will provide quantitative insights of the IAM results. This is also one of the aims of this study.

## Models participating in ICA-RUS and the multi-model simulation procedure

To deal with various uncertainties, four IAMs, i.e., MARIA-14, EMEDA, GRAPE, and AIM, participated in the ICA-RUS project to provide different views and universal information concerning the societal impacts of global warming mitigation strategies. A brief documentation of these models is presented below.

### MARIA-14 (Mori 2000; Mori and Saito 2004; Mori et al. 2013)

MARIA-14 is a monolithic inter-temporal optimization model involving energy technologies, economic activities, land use, food demand–supply systems, and simple climate blocks. The world is disaggregated into 14 regions in ICA-RUS. Economic activity is aggregated into a single sector, and detailed energy flows are involved. MARIA deals with 9 primary energy sources, 3 final energy demand sectors, and 14 fired power generation technologies other than non-biomass renewables and nuclear power. Nuclear power fuel recycling is explicitly included. Non-carbon greenhouse gas (GHG) emissions are provided exogenously.

### EMEDA-MER (Washida et al. 2014; Sakaue et al. 2015)

EMEDA-MER is a monolithic dynamic computable general equilibrium (CGE) model with an iterative optimization procedure. EMEDA includes eight world regions and eight industry sectors, and no energy technology flows are explicitly involved. CO_2_ emissions and global warming are internally generated to explicitly assess the damages of climate change on economic activities as a function of economic activity similar to the DICE model (Nordhaus 1992, 2016). Gaming simulations between subjects are available.

### GRAPE (Kurosawa 2006)

GRAPE is an inter-temporal optimization model involving one aggregated economic activity and 15 world regions. GRAPE includes 12 primary energy sources, 13 energy conversion technologies, and 2 final energy consumption sectors. The latest GRAPE model consists of two sub-models: one is an inter-temporal optimization model, including energy flows, economic activities, and land use changes, and the other represents climate changes including atmospheric and an oceanic carbon circulation. These two sub-models are not simultaneously optimized, but provide solutions by exchanging results.

### AIM/CGE (Fujimori et al. 2012)

AIM is currently an integrated assessment project involving various independent models. AIM provides results by exchanging outputs between models. As part of AIM, AIM/CGE, was developed as an iterative optimization model, dealing with 17 world regions and 43 economic sectors worldwide. AIM includes an energy technology flow model besides AIM/CGE involving 12 primary energy sources, 24 energy conversion technologies, and 5 final energy consumption sectors. A land use change model with agricultural activities included in AIM generates crop production as well as various GHG emission trajectories.

In the ICA-RUS project, the above four IAMs provide data output based on basically the same societal scenarios according to the SSPs (O’Neill et al. 2017). The ICA-RUS project uses the SSP1, SSP2, and SSP3 scenarios as the simulation bases. In our study, since AIM participated in the SSP activity and provided data set for the SSP scenarios (Fujimori et al. 2017), we use the AIM outputs as references. To begin with, we extract AIM-output scenario data concerning population, GDP in market exchange rate, final energy consumption, and GHG emission pathways by country. The parameters of EMADA, GRAPE, and MARIA are then adjusted to harmonize with those of AIM for each SSP in terms of GDP in market exchange rate, final energy consumption, and GHG emissions. Standardized crop yields under SSP-RCP (Sakurai et al. 2014) are also used by MARIA, GRAPE, and AIM. The parameter adjustment procedure may differ between models. For example, MARIA adjusts the autonomous technological progress term in its production functions, final energy demand functions, and some cost parameters of power generation technologies so as to harmonize with the reference data basically giving within 15% ranges of data as BAU.

The above adjustment procedure is used in IPCC-SRES (IPCC 2000). Generally speaking, it is controversial as to whether such adjustment procedures are necessary, because excessive parameter tunings, e.g., too fast productivity growth or too cheap assumptions for future technologies, can harm the internal consistency of the model, resulting in counterintuitive results or calculation instabilities. To avoid this, in this research, the models are carefully checked by the developer as well as other Theme-4 members by the inter-comparison of figures and numbers to exclude unrealistic parameters as possible. Needless to say, we might not have excluded all possible inappropriate assumptions, but the multi-model inter-comparison may have minimized the contamination of the inappropriate assumptions beyond what could be done with single model simulations.

When the base-line parameter calibration for the baseline is completed, the models generate simulation results under the climate policies provided by ICA-RUS Theme 1, i.e., limiting the atmospheric temperature rise to 1.5, 2.0, and 2.5 °C from pre-industry levels. Several additional policy cases described in the following section are also used. By sharing the common projection of population, GDP, CO_2_ emission, final energy consumption on BAU and CO_2_ emission pathways in the climate policy cases, each model generates the simulation results.

**Fig. 3:**
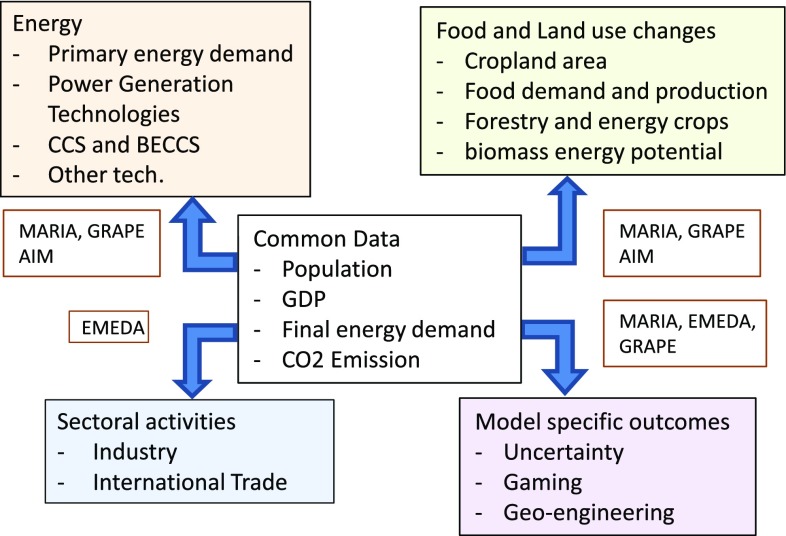
Data supply and expansion structure of the four models

Each model provides common data sets according to the IIASA-model simulation database (IIASA 2017b), including GDP by sector, consumption, primary energy supply by type, final energy demand by type and purpose, implementation of energy conversion technologies with or without carbon capture and storage (CCS), GHG emissions by type, land use, and agricultural production and consumption by type. Although each model also provides additional model-specific information reflecting its unique properties, e.g., geoengineering, adaptation, and economic sectoral impacts, this paper touches upon only the common results for the multi-model inter-comparison. The model-specific results will be described in other papers provided by each model developer. The data generation of the models is shown in Fig. 3.

## Model simulations and results

### Simulation cases

Shared socioeconomic pathways (SSPs) are developed to analyze the feedbacks between climate change and socioeconomic factors, such as world population growth, economic development, and technological progress by the world climate research communities (O’Neill et al. 2017). There are five scenarios in SSPs, i.e., SSP1 (Sustainability—a green road), SSP2 (Middle of the road), SSP3 (Regional rivalry—a rocky road), SSP4 (Inequality—a road divided), and SSP5 (Fossil-fueled development—the highway). The ICA-RUS project selects SSP1, SSP2, and SSP3 from five SSPs for consideration. It is because the carbon emission pathway of SSP4 is similar to that of SSP3. SSP5 is omitted, because the fossil-fueled world seems relatively unlikely when considering the recent rapid expansion of renewable energy sources involving OECD countries, China and India. In fact, some models failed to represent the SSP5-BAU carbon emission pathways in preliminary calculations unless very low technological progress was assumed in the energy-related sectors. On the other hand, because the axis from SSP1–SSP3 via SSP2 represents the sustainable development goal (SDG) dimension, ICA-RUS project considered that the assessment on this axis would be easy for the policy makers to understand.

The calculation of the SSP scenarios without a climate policy is followed by the three climate policy case simulations, where the atmospheric temperature rise in 2100 is limited to no more than 1.5, 2.0, and 2.5 °C under a 3.6 °C equilibrium climate sensitivity, which is slightly higher than the “best estimates,” around 3.0 °C, in IPCC-AR4 (IPCC-AR4-WG1 2007; IPCC-AR4-SPM 2007). ICA-RUS employed 3.6 °C equilibrium climate sensitivity from the view of risk aversion. ICA-RUS applied log-normal distribution to the existing climate sensitivity estimates according to Lewandowski et al. (2014); the project then evaluated so as to achieve the target temperature in 2100 with 66% probability rather than 50% probability. These cases are called T15S36, T20S36, and T25S36, respectively. We also developed five variants on T20S36 for a sensitivity analysis, as presented below.

High potential renewables (HRnws): The energy costs of renewables decrease by 50%, the potential installation capacity is doubled, or both cost reduction and potential capacity expansion occur.

Low Biomass (Lbio): The biomass supply capacity is limited to 80 EJ (MARIA) and 100 EJ (GRAPE) until 2100.

### Low CCS (Lccs): the CCS installation capacity saturates after 2030

*Low Nuclear (Lnuc)* The nuclear power capacity saturates after 2030 (MARIA) and after 2040 (GRAPE).

*CS36–CS45 (Learning)* The world behaves according to T20S36 until 2050 and then suddenly realizes that cumulative carbon emissions until 2100 should be no more than that of T20S45 (a 2.0 °C target under a 4.5 °C climate sensitivity).

**Fig. 4:**
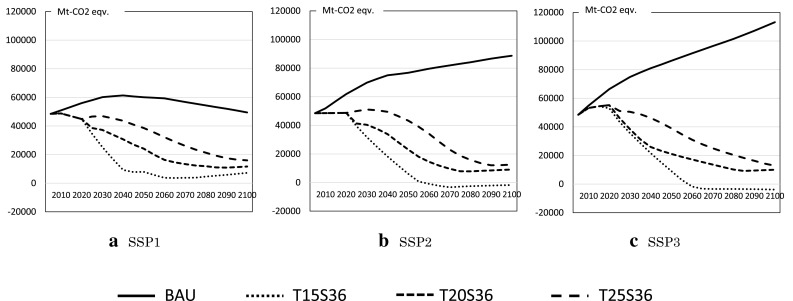
Reference GHG emission pathways for SSP1, SSP2, and SSP3 BAU and climate policy cases in million tons of CO_2_ equivalent to those given by AD-DICE

The reference emission pathways of BAU and climate policy cases are provided by Theme 1 of ICA-RUS based on the expanded AD-DICE model (Su et al. 2017), as shown in Fig. 4. This figure shows that emissions in the base cases spread broadly between the SSPs, and emissions in the climate policy cases show similar patterns. This suggests that the climate control cost of SSP3 would be higher than that of SSP1.

When we calculate the above climate policy cases, MARIA and GRAPE impose constraints on cumulative emissions by 2100 rather than on annual emissions to give a smooth behavior to their dynamic variables, and EMEDA and AIM impose upper limits on annual GHG emissions; this is because two of the four models, MARIA and GRAPE, are inter-temporal optimization models, whereas EMEDA and AIM are iterative optimization models. Therefore, the simulation results of MARIA and GRAPE show slightly different profiles from those of the other models.

In certain scenarios, some models lost feasibility. We summarize the model simulation results, as shown in Table 1.

**Table 1:** Summary of model simulations

	SSP1				SSP2				SSP3			
	MARIA	EMEDA	GRAPE	AIM	MARIA	EMEDA	GRAPE	AIM	MARIA	EMEDA	GRAPE	AIM
Policy cases												
T25S36	Opt.	Opt.	Opt.	Opt.	Opt.	Opt.	Opt.	Opt.	Opt.	Opt.	Opt.	Opt.
T20S36	Opt.	Opt.	Opt.	Opt.	Opt.	Opt.	Opt.	Opt.	Opt.	Opt.	Opt.	Opt.
T15S36	Opt.	n.a	Opt.	Opt.	n.a.	n.a.	Opt.	Opt.	n.a.	n.a.	Opt.	n.a.
T20S36 variants												
High RNWs					Opt.		Opt.					
Low Biomass					Opt.		Opt.					
Low Nuclear					Opt.		Opt.					
Low CCS					Opt.		Opt.					
CS36–CS45							Opt.					

T**S36 in the policy cases represents the case, where atmospheric temperature rise in 2100 is limited at *.* Celsius degree assuming climate sensitivity to be 3.6 Celsius degree

*Opt*. optimum solution obtained, *n.a*. infeasible blank: not calculated

### Simulation results

The regional disaggregations of the models differ from each other. Therefore, for the multi-model comparison, the models aggregate their original regions into five SSP regions, i.e., ASIA (Asian countries), OECD (OECD countries), LAM (Latin American countries), REF (former centrally planned economies), and MAF (Middle East and African countries).

#### CO_2_ emission pathways

**Fig. 5:**
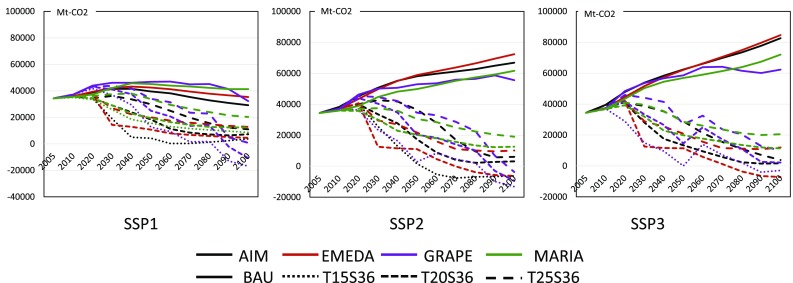
Global CO_2_ emission pathways in millions of tons of CO_2_ for SSP1, SSP2, and SSP3

Figure 5 compares the CO_2_ emission pathways of the four models for the BAU, T15S36, T20S36, and T25S36 cases under the SSP1, SSP2, and SSP3 scenarios. Note that the definitions of CO_2_ emissions differ slightly between models. For example, EMEDA only generates fossil fuel-based CO_2_, whereas AIM and GRAPE take into account emissions from the biosphere and the ocean. MARIA assesses CO_2_ emission from land use changes, but the initial values are set to zero. Therefore, we adjusted the initial values of CO_2_ emissions to those of AIM. Figure 5 shows the CO_2_ emission pathways of the models in the BAU and policy cases for the SSP scenarios.

It can be seen that the CO_2_ emission pathways of the models in the BAU case show similar patterns with an error range of approximately 20%. In the climate policy cases, the CO_2_ emission patterns tend to diverge for different models and regions. The GRAPE model tends to reduce CO_2_ emissions rapidly and emphasizes the need for negative emission options such as biomass-based CCS (BECCS) by the end of this century, whereas EMEDA and MARIA decrease CO_2_ emissions in the first half of this century. In the carbon control strategy cases of SSP3, GRAPE shows non-monotonic trajectories. CO_2_ emissions decrease until 2050, increase once, and then decrease again. This is caused by the increasing population in developing regions and the increasing demand for crop as well as biofuels; this increases the carbon emissions from land use changes, whereas those from fossil fuel demands monotonically decrease throughout the century. Non-monotonous trajectory can be observed because of the complicated interactions between sectors. The regional distribution of carbon-emission reduction depends on various factors, i.e., the economic conditions, primary energy usages, energy saving potentials, land use change potential, and so forth. The behaviors of these factors are shown in the following subsections.

#### GDP losses

**Fig. 6:**
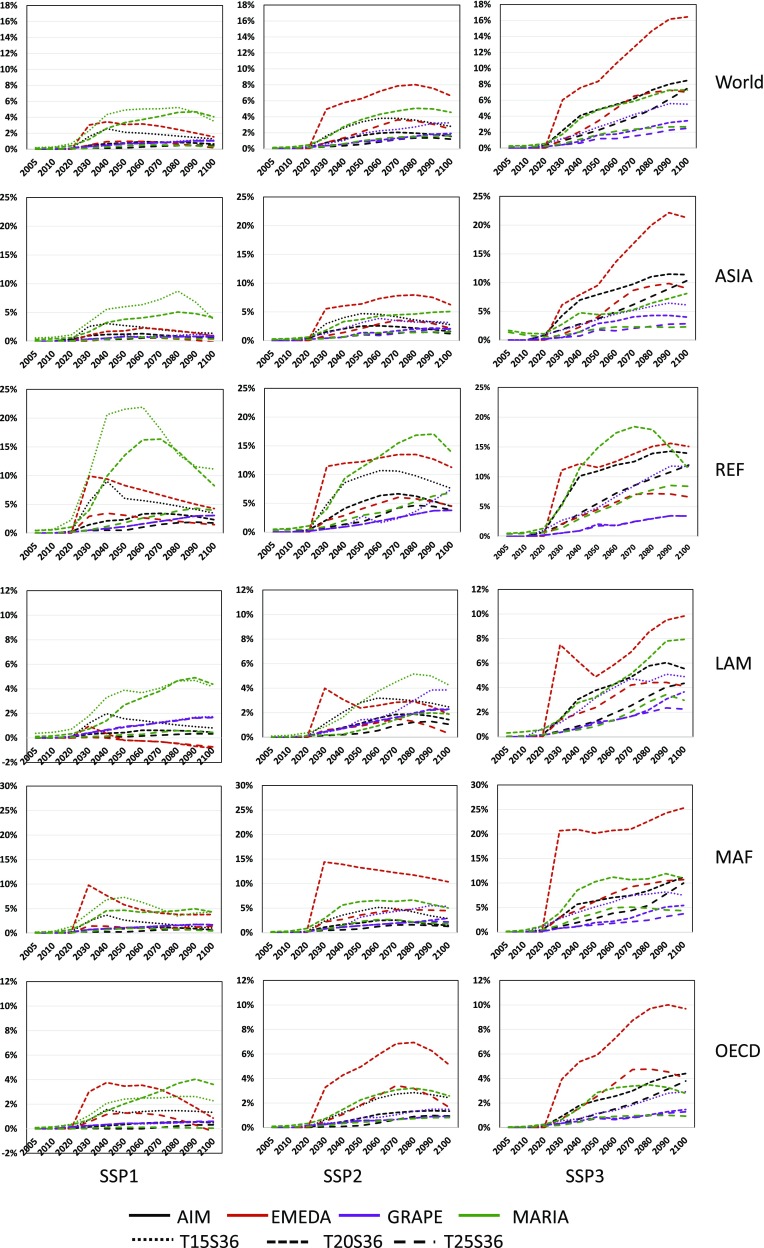
GDP loss in policy cases relative to the BAU for SSP1, SSP2, and SSP3

Here, we focus on the GDP in market exchange rates, because similar conclusions arise from GDP-PPP and consumption. Figure 6 summarizes the rates of GDP decrease from the BAU case.

It is clear that the GDP losses increase rapidly as the climate control target is lowered. The 1.5 °C target case fails to give solutions, except in GRAPE, for all SSPs. Even though the world GDP losses are largely spread between models, they fall within 6% of one another for SSP1 and 8% for SSP2, which are within the ranges of the IPCC picture, as shown in Fig. 2. One can see that EMEDA tends to generate higher GDP losses than the others for SSP2 and SSP3. GDP losses in OECD and LAM are slightly lower than those in other countries. It is remarkable that the GDP loss in the MAF and REF regions, where fossil fuel resource endowment is high, is much higher than those in other regions in all carbon control cases, except those in GRAPE. GRAPE introduces significant CCS options, as suggested in Fig. 5. Therefore, the economic losses caused by climate policies can be mitigated if large amounts of CCS and biomass are available. The possible relationships between the economic loss and the technology options in the climate policy cases are discussed further in Sect. 3.3.

#### Final energy consumption

**Fig. 7:**
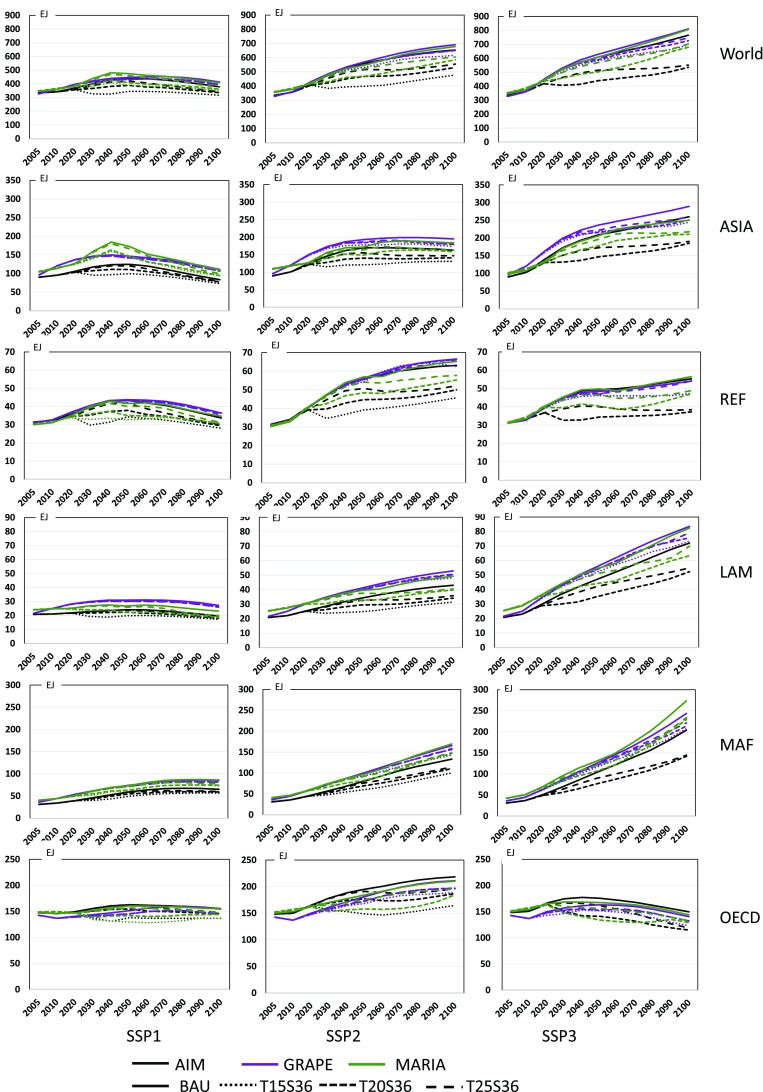
Final energy consumption in the BAU and policy cases in EJ for SSP1, SSP2, and SSP3

The final energy consumption is provided by MARIA, GRAPE, and AIM. Figure 7 shows how the final energy consumption decreases when the stringent climate policy is applied. One can observe that the final energy consumption patterns in the BAU case basically agree between models. In all regions, final energy consumptions should decrease significantly in all SSPs, but the decline rate in SSP3 is much larger than that of SSP1. It suggests that the energy service demand in SSP3 would seriously be cut under climate policy cases. Note that the final energy demand conservation in AIM appears to be more optimistic than those of the other models in all regions. The assessments of the potentials and the costs of energy conservation are controversial but critically important to climate policy.

#### Primary energy supply

**Fig. 8:**
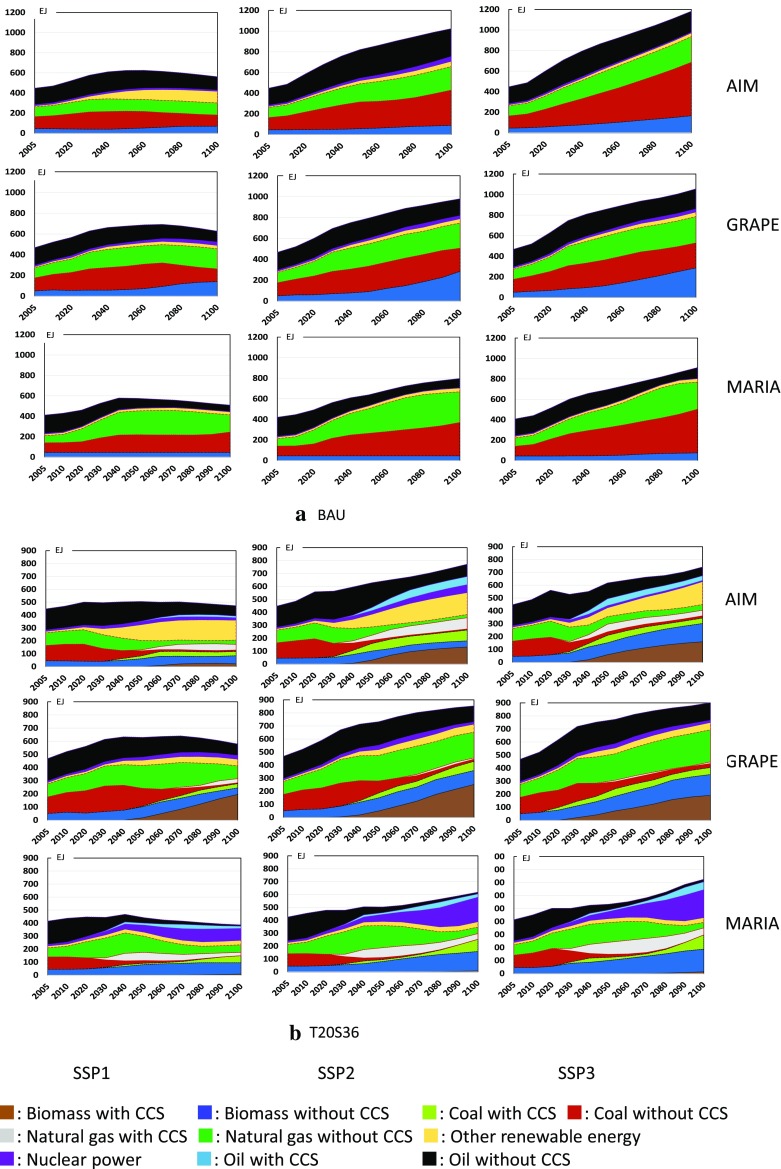
Worldwide primary energy mix in BAU and T20S36 for SSP1 (left), SSP2 (middle), and SSP3 (right) by model; AIM (upper), GRAPE (middle), and MARIA (bottom)

**Fig. 9:**
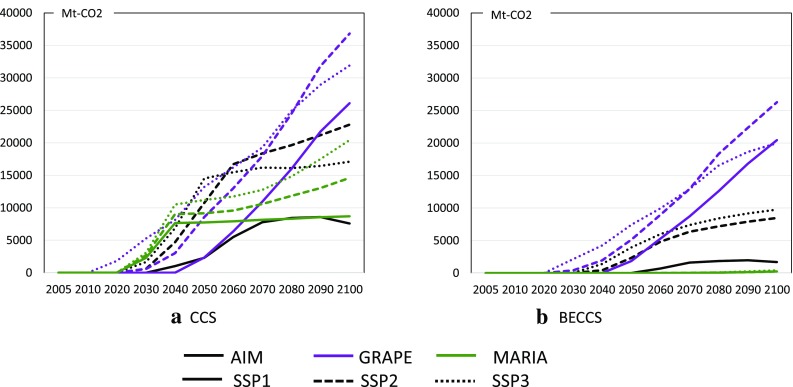
World CCS and BECCS implementation in T20S36 of AIM, GRAPE, and MARIA for SSP1, SSP2, and SSP3

Energy mixing is a key factor in climate policy. Figure 8 compares the results of three models for the world total in the BAU and the T20S36 policy cases. Figure 9 focuses on the CCS and BECCS implementation patterns among the models and SSPs. In the BAU case, all models basically show similar patterns, where conventional fossil fuels, especially coal and natural gas, are still mainly used, whereas GRAPE shows increasing biomass demand. Note that nuclear power is negligibly small in all models and cases. The characteristics of the models can be clearly observed in the climate policy case. AIM introduces significant amounts of renewable energy sources, i.e., hydropower, wind, and solar power, and then, biomass with CCS is used. GRAPE implements significant amounts of CCS in all scenarios, whereas MARIA prefers nuclear power and fossil fuels with CCS. MARIA deploys significant amounts of biomass but not BECCS. Neither GRAPE nor MARIA is optimistic about the expansion of renewable energy compared with AIM. Therefore, the primary energy mix tends to diverge for the different models. In other words, there are still various technological alternatives that can achieve the same climate targets; however, biomass and CCS are essentially used in all the models.

#### Power generation mix

**Fig. 10:**
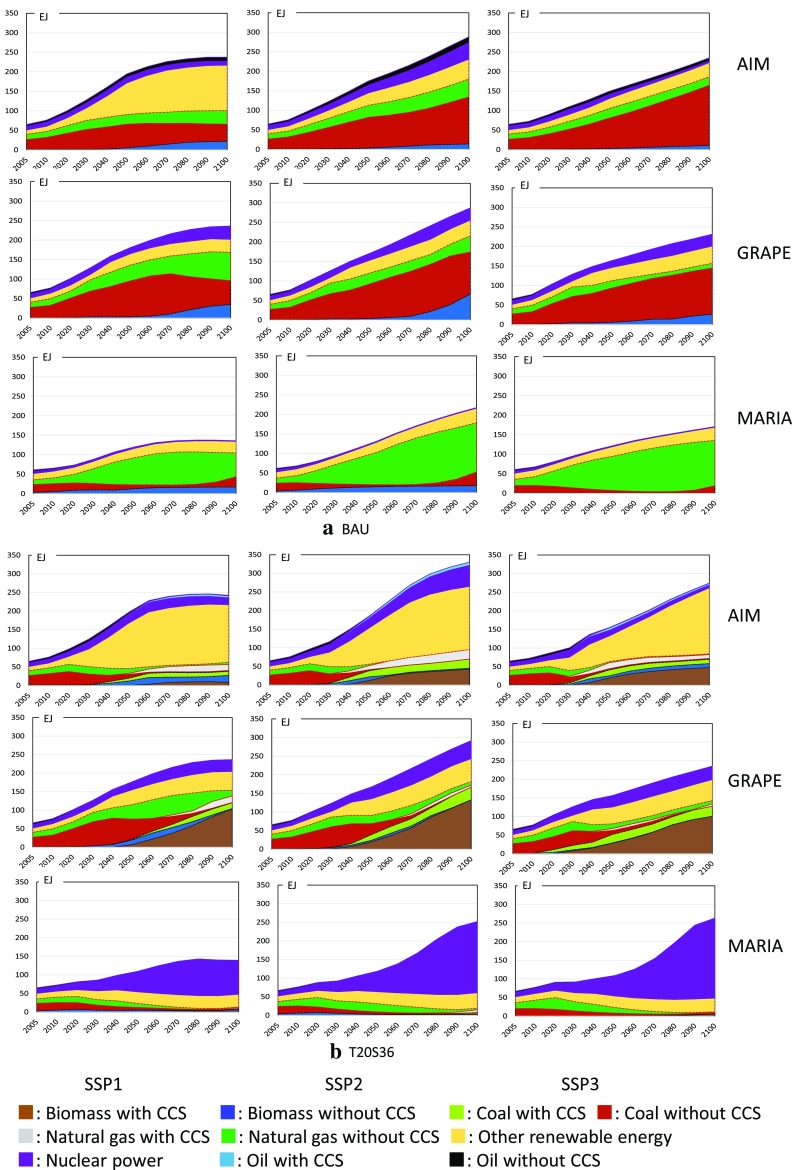
Worldwide power generation mix in BAU and T20S36 for SSP1 (left), SSP2 (middle), and SSP3 (right). The first, second, and third row graphs show results of AIM, GRAPE, and MARIA, respectively

Model characteristics are further emphasized when we look at the power generation mix, because biomass fuels are primarily used in the transportation sector. Figure 10 shows the inter-model comparison in BAU and T20S36 cases. This figure shows the differences of future power generation pathways between models clearly. All models show the large share of fossil fuels in BAU, whereas the key sources are slightly different between models. In SSP1, AIM shows high share of non-biomass renewables and coal, while natural gas is the main source in MARIA in contrast to GRAPE, where coal is the major source. In SSP1, MARIA generates relatively low power generation than other two models. In T20S36 case. AIM depends on non-biomass renewable energy sources, whereas MARIA depends on nuclear power. Nuclear power expansion is often limited exogenously depending on societal acceptance, whereas the MARIA simulation includes the maximum technological potential of nuclear fuel recycling. GRAPE apparently depends on BECCS. This figure allows society to compare future alternative energy strategies.

#### Crop production and land use change

**Fig. 11:**
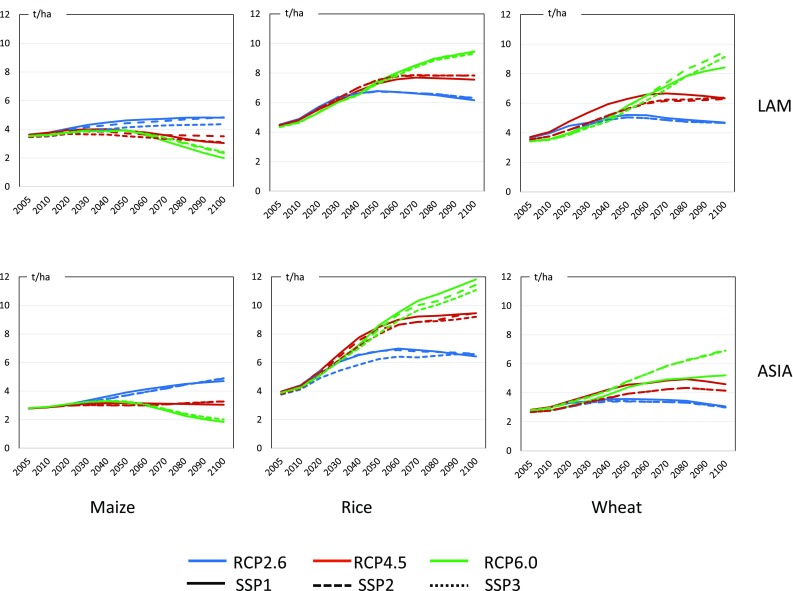
Example of regional crop yield projections for maize (left), rice (middle), and wheat (right) and LAM (upper) and ASIA (lower) in SSP-RCPs in ton per hectare (Sakurai et al. 2014)

**Fig. 12:**
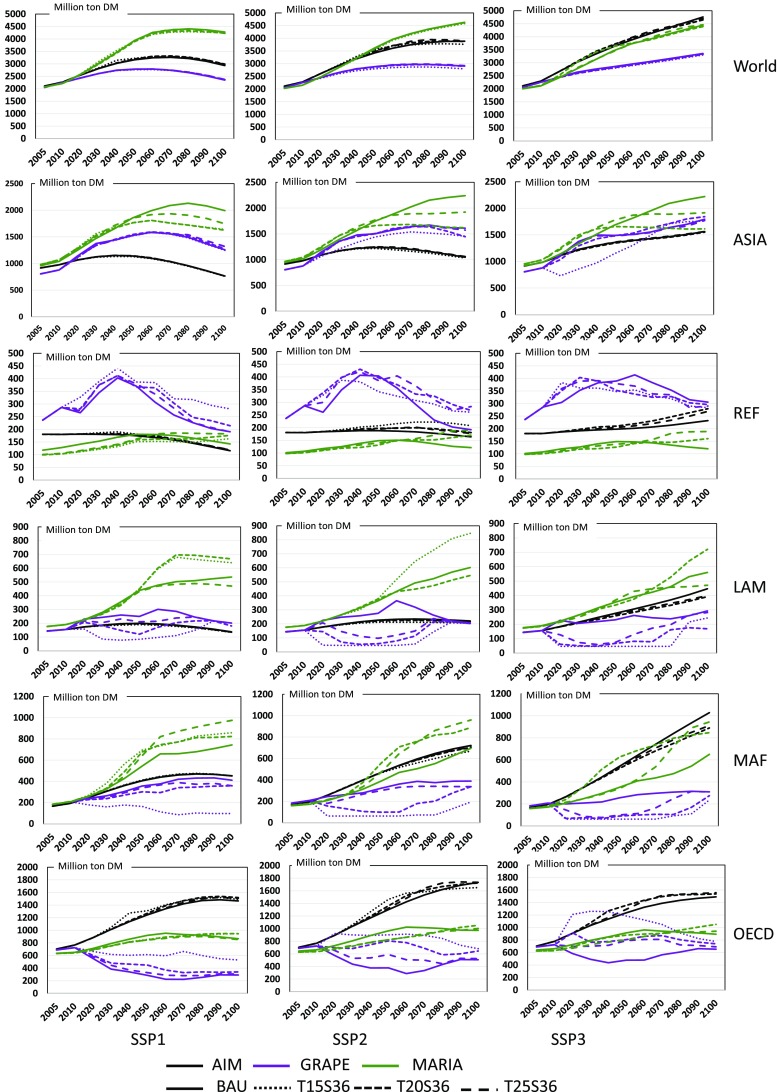
World and regional crop production under the BAU and climate policy cases for SSP1 (left), SSP2 (middle), and SSP3 (right)

The projected crop production and the land use changes are strongly related to competition between the biomass utilization potential and the sustainability of society. Projection of the crop yield under climate changes is provided by ICA-RUS Theme 1, where the yields of maize, soybeans, spring wheat, winter wheat, and rice are provided by country and by climate change scenario in SSP–RCP. Figure 11 shows several examples. This figure suggests that yields are strongly affected by SSP scenarios and crop types, whereas changes in the yield between policy cases are small. We aggregated regional yields according to the model-specific region (Sakurai et al. 2014). Figure 12 shows the results of total crop production. Because crop types included in the model differ between models, it is not possible to directly compare the model simulation results. Nonetheless, it can be observed that the world crop production does not change significantly for the different climate policy cases, whereas the crop yields change depending on the regional and climate policies. Even though the world production is nearly constant throughout the different climate policy cases, regional production is strongly affected by the climate policy in all the models. These figures also show the apparent regional difference between models. For instance, AIM shows small differences between SSP scenarios and climate policy cases. MARIA also such shows small changes in REF and OECD, whereas in ASIA, LAM, and MAF, crop production varies between climate policy cases. GRAPE generates remarkable changes among climate policy cases. The OECD region produces more crops when climate policy is introduced, whereas LAM and MAF decrease crop production in all SSPs. As shown in Figs. 8 and 9, GRAPE implements biomass and BECCS largely under climate policies. Those differences reflect the differences in the need for energy crops and BECCS among models. Note that the assumptions in the potential crop-land availability for food production and demand differ between the models. Because land use changes and food demand–supply projection are essential for seeing the potential contributions of energy crops as well as the social sustainability, especially in the stringent climate policy cases, this point should be investigated further.

#### T20S36 variants

The MARIA and GRAPE models provide results for the five T20S36 variants. Table 2 summarizes the changes in GDP losses for GRAPE and MARIA. The sensitivity analysis clearly shows the properties of the models. In GRAPE, the effects of CCS are greatest, causing an approximately 2.5% decrease in GDP for the world. The losses in the ASIA, REF, and MAF regions are much larger than those in the other regions, whereas the effects of high potential renewables and nuclear power are small. The MARIA simulations appear to be more sensitive than those of GRAPE; the effects of CCS are not large; however, nuclear power constraints and biomass constraints cause high GDP losses, especially in the REF region.

**Table 2:** Comparison of GDP losses of T20S36 variant cases

Region	Year	GRAPE				MARIA			
		High potential renewables	Low biomass	Low CCS	Low nuclear	High potential renewables	Low biomass	Low CCS	Low nuclear
		T20S36_HRnws (%)	T20S36_Lbio (%)	T20S36_Lccs (%)	T20S36_Lnuc (%)	T20S36_HRnws (%)	T20S36_Lbio (%)	T20S36_Lccs (%)	T20S36_Lnuc (%)
World	2030	0.00	0.00	0.30	0.00	0.00	0.00	0.00	0.10
World	2050	− 0.10	0.30	0.80	0.00	− 0.20	0.00	0.20	0.10
World	2100	− 0.10	0.20	1.20	0.00	− 0.60	0.10	0.40	0.30
OECD	2030	0.00	0.00	0.00	0.00	0.00	0.00	0.00	0.00
OECD	2050	− 0.10	0.10	0.00	0.00	− 0.10	0.00	0.00	0.10
OECD	2100	0.00	0.00	0.20	0.00	− 0.30	0.10	0.10	0.20
ASIA	2030	0.00	0.00	1.10	0.00	0.00	0.00	0.10	0.10
ASIA	2050	− 0.20	0.70	1.30	0.10	− 0.20	0.00	0.00	0.10
ASIA	2100	0.00	0.60	1.90	0.00	− 0.60	− 0.10	0.00	0.20
REF	2030	0.00	0.00	0.00	0.00	− 0.20	0.10	− 0.50	0.20
REF	2050	0.00	0.00	0.70	0.00	− 0.40	0.20	2.00	0.50
REF	2100	0.00	0.00	1.40	0.00	− 2.10	− 0.10	3.10	0.70
LAM	2030	0.00	0.00	0.00	0.00	− 0.10	0.00	0.00	0.10
LAM	2050	0.00	0.00	1.10	0.00	− 0.20	0.10	0.00	0.20
LAM	2100	0.00	0.10	0.30	0.00	− 0.60	0.00	0.30	0.40
MAF	2030	0.00	0.00	0.00	0.00	− 0.10	0.00	0.10	0.20
MAF	2050	0.00	0.20	2.00	0.00	− 0.20	0.10	0.40	0.30
MAF	2100	− 0.20	0.00	2.20	0.20	− 0.60	0.20	0.80	0.60

High Potential Renewables (HRnws): The energy costs of renewables decrease by 50% and the potential installation capacity is doubled

Low Biomass (Lbio): The biomass supply capacity is limited to 80 EJ (MARIA)/100 EJ (GRAPE) until 2100

Low CCS (Lccs): The CCS installation capacity saturates after 2030

Low Nuclear (Lnuc): The nuclear power capacity saturates after 2030 (MARIA)/after 2040 (GRAPE)

**Fig. 13:**
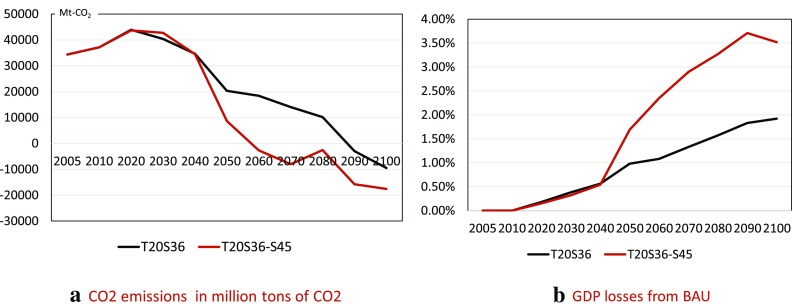
Comparison of T20S36 and S36–S45 for CO_2_ emissions and GDP losses of GRAPE

For the “learning case,” in which the decisions before 2050 are set based on T20S36 and cumulative carbon emissions by 2100 are suddenly lowered when recognizing that the correct climate sensitivity is 4.5 °C, only GRAPE can provide results. Figure 13 visualizes the CO_2_ emissions and GDP losses to see the effects of the learning case. The GDP losses of the learning case increase rapidly after 2050. It is remarkable that the GDP at the end of this century for the S36–S45 case is nearly the same as that for T15S36, as shown in Fig. 6. This observation suggests that postponing climate policy actions will cause large economic losses to future generations.

### Meta-analysis of the multi-model results: are the model results consistent?

The above multi-model inter-comparison based on harmonized assumptions yields agreement in observations and projection ranges. The question is whether these model results are consistent. When differences are caused by the model specification, more effort should be expended on model internal consistency or structural issues. Conversely, if we can find a consistent relationship in the models, we should examine the cost assumptions and technology potentials.

The meta-analysis based on statistical analysis of the database would provide deeper insights; however, such an analysis has not yet been successfully reported.

In the ICA-RUS project, because the model parameters are calibrated according to the SSP scenarios, we expect statistical analysis to provide deeper insights into the structures of the models.

In this study, we first focused on the world GDP losses (GDP-L) in Fig. 6 to explore the consistent relationships between the models and scenarios.

First, we picked the candidates for the explanatory variables as follows: (1) CO_2_ emission reduction rates from the BAU case (CO_2_-L); (2) final energy demand reduction rates from the BAU case (FE-L); (3) CCS implementation in Gt-CO_2_ (CCS); (4) biomass energy consumption in EJ (BIO); (5) nuclear power in EJ (NUC); (6) non-biomass renewables in EJ (RNW); and (7) the lagged GDP losses [GDP-L(− 1)].

Table 3 shows the correlation coefficients of the variables of the three models and the three policy cases for 2010–2100. GDP-L(− 1) gives the highest correlation with GDP-L. One can also observe a high correlation between CCS and CO_2_-L. Because most variables are positively correlated, we need to be careful when selecting the regression structure to avoid multi-collinearity.

**Table 3:** Correlation coefficients between variables

	GDP-L	CO2-L	FE-L	CCS	BIO	NUC	RNW	GDP-L(-1)
SSP1								
GDP-L	1.000							
CO2-L	0.545	1.000						
FE-L	0.692	0.605	1.000					
CCS	0.271	0.871	0.205	1.000				
BIO	0.196	0.751	0.011	0.906	1.000			
NUC	0.836	0.464	0.449	0.261	0.196	1.000		
RNW	0.026	0.437	0.656	0.163	0.014	− 0.140	1.000	
GDP-L(− 1)	0.958	0.575	0.657	0.305	0.221	0.875	0.079	1.000

	GDP-L	FE-L	CO2-L	CCS	BIO	NUC	RNW	GDP-L(− 1)
SSP2								
GDP-L	1.000							
FE-L	0.447	1.000						
CO2-L	0.690	0.702	1.000					
CCS	0.407	0.409	0.870	1.000				
BIO	0.397	0.225	0.770	0.875	1.000			
NUC	0.778	0.334	0.520	0.302	0.232	1.000		
RNW	0.136	0.720	0.572	0.447	0.432	0.005	1.000	
GDP-L(− 1)	0.971	0.442	0.701	0.446	0.456	0.852	0.192	1.000

	GDP-L	CO2-L	FE-L	CCS	BIO	NUC	RNW	GDP-L(− 1)
SSP3								
GDP-L	1.000							
CO2-L	0.819	1.000						
FE-L	0.642	0.695	1.000					
CCS	0.644	0.899	0.519	1.000				
BIO	0.587	0.754	0.388	0.850	1.000			
NUC	0.679	0.513	0.232	0.419	0.169	1.000		
RNW	0.429	0.564	0.614	0.562	0.701	-0.158	1.000	
GDP-L(− 1)	0.980	0.790	0.584	0.658	0.600	0.726	0.409	1.000

(1) CO_2_ emission reduction rates from the BAU case (CO_2_-L), (2) final energy demand reduction rates from the BAU case (FE-L), (3) CCS implementation in Gt-CO_2_ (CCS), (4) biomass energy consumption in EJ (BIO), (5) nuclear power in EJ (NUC), (6) non-biomass renewables in EJ (RNW), and (7) the lagged GDP losses (GDP-L(− 1))

The final regression equations obtained after eliminating the non-significant variables are as follows, where the *t* values appear in parenthesis.


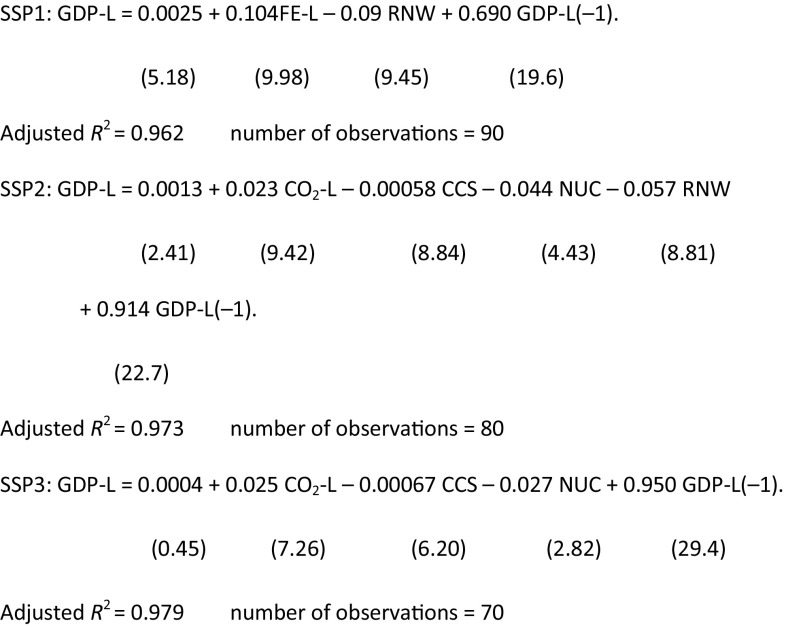


**Fig. 14:**
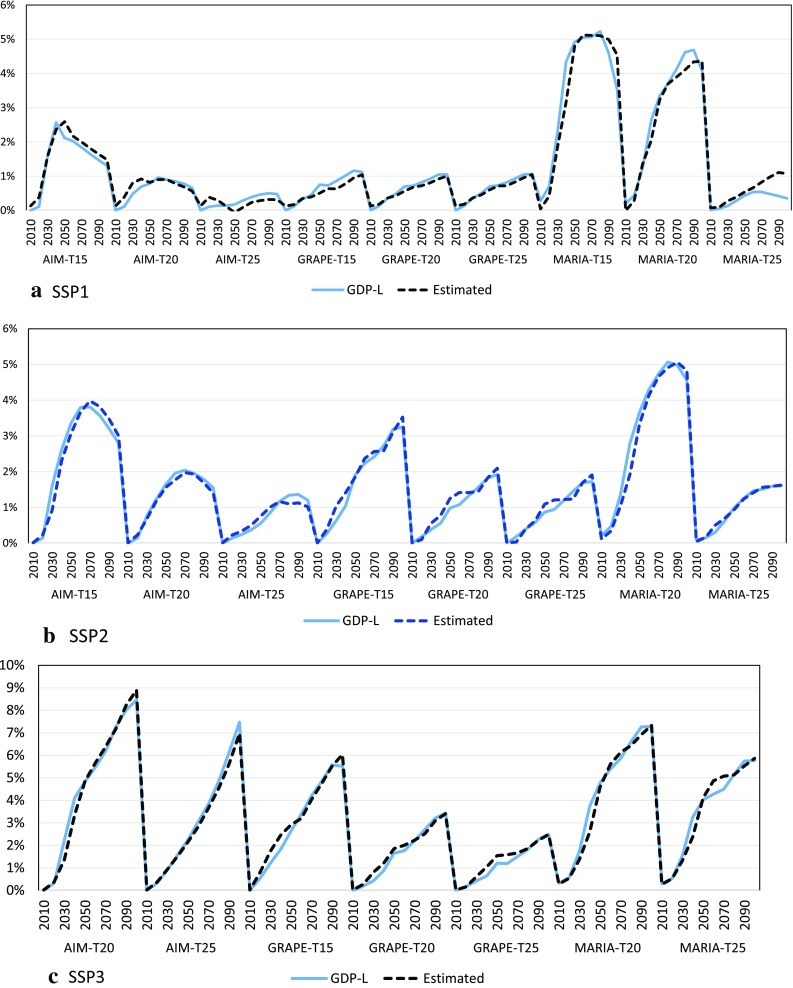
Model simulation results (GDP-L) and estimated values of GDP losses (Estimated) for SSP1, SSP2, and SSP3 The horizontal axis of each model-scenario block represents the years between 2010 and 2100

The regression equations represent slightly different structures of the SSPs. For example, CO_2_-L is eliminated in SSP1, while the contributions of CCS and NUC for the mitigation of economic loss are statistically significant in SSP2 and SSP3. Note that the above equations include no model-specific variables such as dummy variables. Figure 14 shows the original and estimated series of the GDP losses by time, model, and policy cases. These figures show high fitness on the dynamic behavior of the regressions through models and policy cases in all SSPs. These regression equations and figures suggest a consistent structure through the models and climate policy scenarios depending on the SSP scenarios.

Next, we look at the regional data. In this study, we examine the SSP2 policy case. The regression results summarized in Table 4 suggest that the regional GDP losses are well explained by the world total regression equations without model-specific variables. The model structures differ slightly for different regions, and nuclear power and renewables mitigate the GDP loss in certain regions. The coefficients of CCS and biomass are not statistically significant. This is due to the high correlations between CO_2_ reduction and other variables.

**Table 4:** Regression results in SSP2 through policy cases and models

	OECD	ASIA	LAM	MAF	REF	World
FE-L	0.0575	0.0879	0.0727	0.0752	0.231	
*t* value	12.7	9.14	9.99	9.99	12.0	
CO2-L	0.0012			0.00749	0.00993	0.023
*t* value	2.39			2.97	4.55	9.42
BIO			0.00021			
*t* value			5.57			
CCS						− 0.0006
*t* value						8.84
NUC	− 5E−05	− 0.0001	− 0.0017	− 0.0003	− 0.0026	− 0.044
*t* value	2.44	3.6	5.73	5.86	5.75	4.43
RNW	− 0.001	− 0.0003	− 0.0013	− 0.0006	− 0.0026	− 0.057
*t* value	8.18	8.89	10.25	9.02	9.11	8.81
GDP-L(− 1)	0.674	0.873	0.952	0.859	0.812	0.914
*t* value	18.48	20.49	18.48	19	27.6	22.7
Cont	0.0018	0.00369	0.004	0.00259	0.00982	0.0013
*t* value	5.8	6.39	6.03	3.03	6.42	2.41
Adjusted *R* ^2^	0.985	0.956	0.972	0.953	0.975	0.973
num. of obs	80	80	80	80	80	80

We can conclude that the model simulations give a consistent context, even though the models are developed independently and have different structures.

## Conclusions

In this paper, we have described the research outcomes of ICA-RUS Theme 4 focusing on the multi-model approach. On the basis of simulations, we have concluded the following. First, for the stringent climate target, the regional economic losses of the models tend to diverge, whereas the global total economic loss does not. Second, CCS and BECCS are essential for making the stringent climate target feasible, even if the deployment potential varies between models. Third, the models show small changes in the world total crop production and large differences between individual regions. Fourth, the meta-analysis shows a consistent relationship through models, even if the model structure and technological assumptions differ. Because this is just a preliminary exercise in statistical meta-analysis including three models and three policy cases, it is expected that more sophisticated methods to deal with the existing large database on IAM simulation results, including data mining or machine learning, will be able to extract the implicit information behind the models.

We conclude that our multi-model research activities have generated a possible and consistent variety of options under the uncertainties in climate policy targets, technology potential, and societal projections. Although the uncertainties we have dealt with are limited, we hope that our outcome contributes to the risk management context of long-term decision making.
